# m6A RNA methylation in neural plasticity, brain aging, and neurodegenerative vulnerability

**DOI:** 10.1186/s13041-026-01297-z

**Published:** 2026-03-29

**Authors:** Xuehua Zhou, Peiyang Yu, Xia Shen

**Affiliations:** 1 Department of Anesthesiology, Eye & ENT Hospital, Fudan University, 83 Fenyang Road, Shanghai, China; 2 Huashan Clinical Medical College, Shanghai Medical College, Fudan University, Shanghai, China

**Keywords:** m6A RNA methylation, Neural development, Aging, Experience-dependent plasticity, Neurodegenerative diseases

## Abstract

m6A is a pervasive post-transcriptional RNA modification that regulates RNA splicing, stability, localization, and translation in the brain. In this review, we outline the core m6A regulatory machinery and summarize its spatial organization across neurons and glial cells, highlighting established roles in brain development, synapse formation, and axon growth. We then focus on experience-dependent plasticity, synthesizing evidence that neuronal activity and environmental inputs dynamically reshape m6A to regulate immediate-early transcription and local translation at synapses across sensory, cognitive, emotional, and motor domains. With aging, m6A programs are reconfigured in a cell-type–specific manner, a shift associated with reduced plasticity and increased vulnerability. We further survey disease-associated alterations in m6A across Alzheimer’s disease, Parkinson’s disease, Huntington’s disease, stroke-related cognitive impairment, ALS and FTD, as well as metal or toxin exposure, emphasizing convergent effects on dopaminergic and glutamatergic signaling, synaptic integrity, inflammation, and cellular stress responses. Finally, we discuss emerging opportunities and conceptual challenges in targeting m6A enzymes or reader proteins, and outline priorities for future work, including cell-type– and subcellular-resolved mapping, causal perturbation in defined circuits and life stages, and the development of biomarkers and selective modulators. Together, these observations position m6A as a molecular interface linking experience-dependent plasticity, brain aging, and neurodegenerative vulnerability.

**Graphical abstract:**

**Figure:**
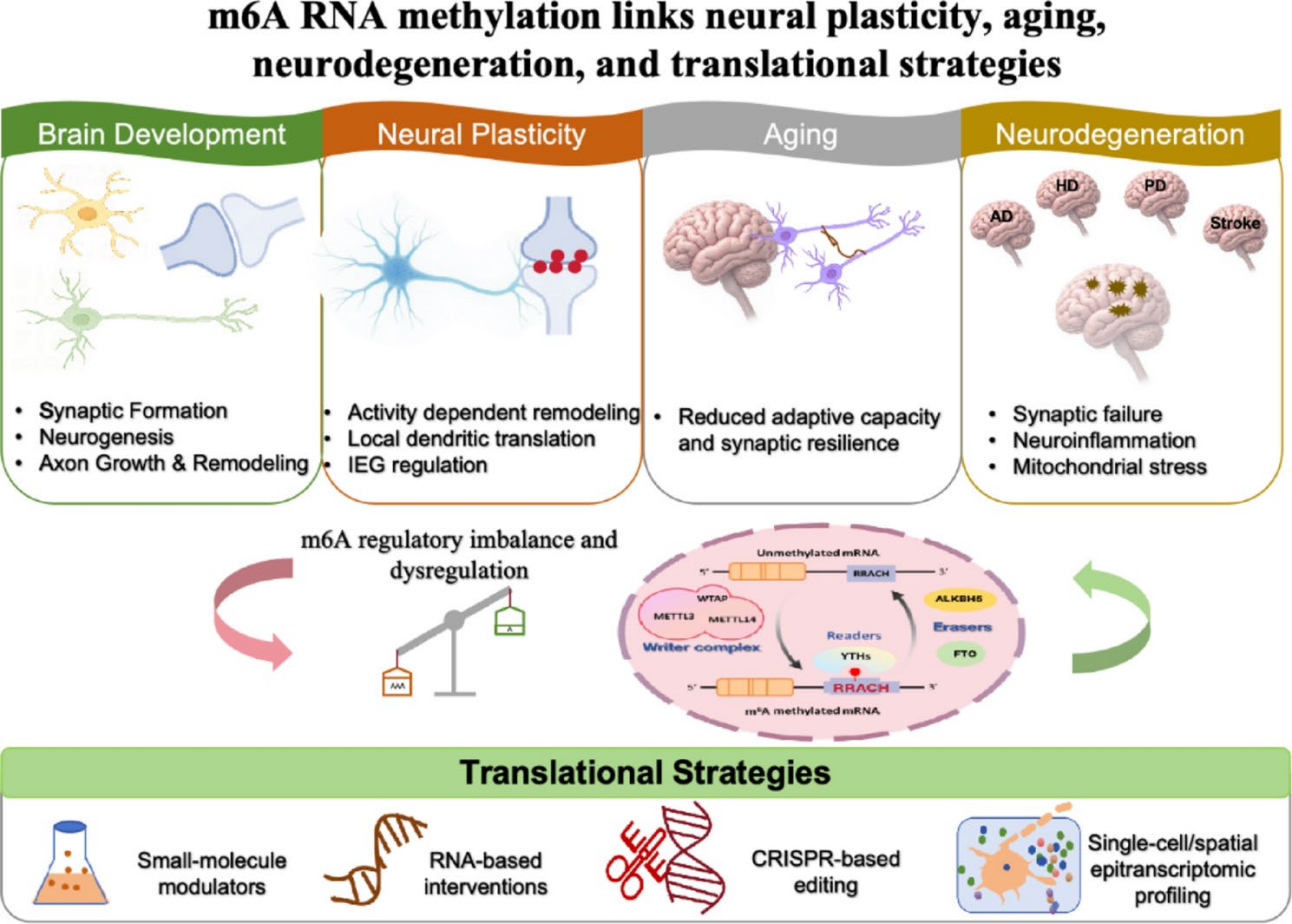
This schematic summarizes m6A RNA methylation as a regulatory interface linking brain development, neural plasticity, aging-related reconfiguration, and neurodegenerative vulnerability. Across these contexts, coordinated actions of m6A writers, erasers, and readers shape RNA stability, translation, and cellular adaptation, whereas dysregulation may contribute to synaptic dysfunction, inflammatory imbalance, mitochondrial stress, and disease susceptibility. The figure also highlights emerging translational avenues, including small-molecule enzyme modulators, RNA-targeted interventions, CRISPR-guided epitranscriptomic editing, and single-cell/spatial epitranscriptomic profiling.

## Introduction

The brain adapts continuously to its environment through dynamic reorganization of neural structure and function, a process that ultimately shapes behavior. Experience-dependent plasticity enables neural circuits to adjust in response to activity patterns and sensory inputs, providing the cellular basis for learning and memory [1]. At the molecular level, these adaptations are supported by activity-dependent gene regulatory programs that allow neurons to rapidly activate or repress transcription in accordance with contextual demands [2, 3].

Epigenetic regulation is central to this form of neural flexibility. DNA methylation, histone modifications, RNA modifications, and nucleosome remodeling collectively influence chromatin accessibility, neuronal differentiation, and synaptic development, thereby linking experience to enduring changes in circuit function [4, 5]. Among RNA-based regulatory mechanisms, N6-methyladenosine (m6A) has emerged as the most abundant and dynamically regulated internal modification in eukaryotic RNA. m6A modulates multiple aspects of RNA metabolism, including stability, translation, and splicing, positioning it as a versatile regulator of gene expression [6, 7]. Its pronounced enrichment in the brain is associated with key neuronal processes, including dopaminergic signaling, neurogenesis, and axonal regeneration [8–10]. Consistent with this view, alterations in m6A levels have been linked to brain development, learning, and memory formation, implicating this modification in neural plasticity [10, 11].

In this review, we first outline the roles of m6A in brain development and physiological function. We then examine how m6A is associated with experience-dependent plasticity across sensory processing, learning, motor behavior, and environmental modulation. Finally, we discuss how disruption of m6A regulation has been linked to disease-related mechanisms, with particular emphasis on neurodegenerative and cognitive disorders, and consider the emerging relevance of targeting m6A regulatory machinery in translational contexts [12, 13]. Through this framework, we aim to bridge fundamental neurobiology and translational neuroscience by positioning m6A as a molecular interface through which experience is associated with neural plasticity and disease vulnerability.

## Dynamic and reversible RNA m6A methylation

RNA methylation was first described in the 1970s, yet its biological significance remained largely unexplored for decades because of technical limitations in detection. Recent advances in high-throughput, transcriptome-wide mapping approaches have transformed this field, enabling precise identification of N6-methyladenosine (m6A) and revealing its widespread involvement in regeneration, cancer, neurodevelopment, and nervous system function [12]. m6A is now recognized as the most abundant and evolutionarily conserved internal RNA modification in eukaryotes, including mammals [14].

m6A is distributed across multiple RNA species, including messenger RNA, microRNA, long noncoding RNA, and ribosomal RNA, where it contributes broadly to post-transcriptional gene regulation [15–18]. A defining feature of m6A regulation is its dynamic and reversible nature. Dedicated methyltransferase complexes (“writers”) install the modification, demethylases (“erasers”) remove it, and m6A-binding proteins (“readers”) decode the mark to influence RNA fate [19]. Through the coordinated actions of these effectors, m6A is associated with regulation of RNA splicing, stability and decay, subcellular localization, and translation, thereby shaping gene-expression programs relevant to neuronal function.

Consistent with this regulatory versatility, disruption of m6A machinery has been linked to altered brain development, impaired memory processes, and a range of neurological disorders [20–22]. Together, these observations support the view that m6A represents a flexible regulatory layer through which RNA metabolism is tuned to developmental and physiological demands. The principal m6A writers, erasers, and readers, along with their core functions and disease associations, are summarized in Table 1.

**Table 1:** Functional roles and disease associations of key m6A writers, erasers, and readers

Category	Name	Function	Disease/Biological associations
Writers	METTL3	Core methyltransferase catalyzing m6A formation; utilizes S-adenosylmethionine (SAM)	Cancers [23, 24] Neurodegenerative diseases (such as Alzheimer’s disease, Parkinson’s disease, Huntington’s disease, Metal-induced cognitive dysfunction) [25–27] Mental health disorders (such as depression and anxiety) [28] Seizure [29]
	METTL14	Recognizes methylation substrates, forms a heterodimer with METTL3 for higher catalytic activity	
	WTAP	Recruits METTL3 and METTL14 to mRNA targets, maintains m6A levels	Brain arteriovenous malformation (bAVM) [30]
	KIAA1429, METTL16, METTL5, and RBM15	Regulates m6A modification on specific mRNAs	Lung cancer [31] Neurological disorders, particularly those affecting motor function and behavior [32]
Erasers	FTO	m6A demethylase; involved in DNA damage repair, heart failure, obesity, neurodevelopment, and regeneration; reduced activity under hypoxic conditions	Cancer [33] Neurological disorders and neurodegenerative diseases [21, 33–37], Obesity and metabolic disorders (such as type 2 diabetes) [21, 33, 34, 38] Ischemic brain injury [21, 23, 33] Cardiovascular diseases [39, 40]
	ALKBH5	Directly converts m6A to adenosine without intermediates	Cancers [33, 34] Neurological disorders (such as Alzheimer’s disease, Parkinson’s disease) [33, 41] and cognitive dysfunction [9, 42, 43] Male infertility, [33, 34] Depression [44] Brain development [45]
Readers	YTHDF1	Enhances translation by recruiting translation initiation factors	Cancer [34] Neurological and cognitive disorders (such as Alzheimer’s disease, Age-related cognitive decline, and Metal -induced cognitive dysfunction) [46–49] Retinal neurogenesis [50, 51] Male infertility [52] Seizure [29]
	YTHDF2	Promotes mRNA degradation	
	YTHDF3	Enhances translation in cooperation with YTHDF1	
	YTHDC1	Regulates mRNA splicing, promotes nuclear export, accelerates RNA degradation	
	YTHDC2	Maintains mRNA stability, regulates translation	
	IGF2BP	Stabilizes target mRNAs	Cancers (such as breast and colorectal cancer) [53] Metabolic disorders (such as type 2 diabetes) [53] Recognition impairment [54–56]
	PRRC2A	Stabilizes m6A-modified transcripts, essential for myelination	Myelination [57]
	hnRNPs	Mediate neuronal mRNA splicing, stability, and transport	Synaptic plasticity and neurodevelopment [58]

### Methyltransferases (“writers”)

Installation of m6A is mediated by a conserved multicomponent methyltransferase complex centered on METTL3, METTL14, and WTAP. METTL3 serves as the catalytic core, containing the S-adenosylmethionine–binding pocket required for methyl group transfer [28]. METTL14 forms a stable nuclear heterodimer with METTL3 and primarily contributes to RNA substrate recognition and structural stabilization, thereby enhancing the efficiency and specificity of methylation [59]. WTAP functions as a noncatalytic regulatory subunit that facilitates proper localization and recruitment of the METTL3/METTL14 complex to target transcripts and is required for maintaining global m6A levels [30].

Beyond the core complex, additional factors refine m6A deposition by modulating writer activity and site selection. KIAA1429 (also known as VIRMA), together with RBM15, RBM15B, and related adaptor proteins, directs methylation toward specific transcript regions or defined RNA contexts, contributing to spatial patterning of m6A marks [38, 60]. Other methyltransferases, including METTL16 and METTL5, act on selected RNA substrates and extend the repertoire of m6A and related modifications within the broader epitranscriptomic landscape [34]. Collectively, these components coordinate the timing and positioning of m6A installation, establishing a flexible layer of post-transcriptional regulation relevant to the complex gene-expression demands of the nervous system.

### Demethylases (“erasers”)

To date, only two enzymes have been definitively validated as m6A demethylases: FTO and ALKBH5, both members of the AlkB family of Fe(II)/α-ketoglutarate–dependent dioxygenases [33]. FTO was originally identified in 1999 as an obesity-associated gene and was subsequently shown to possess m6A demethylase activity [38, 60]. Structurally, FTO contains a C-terminal domain with a folding pattern distinct from other AlkB proteins, a feature proposed to support protein–protein interactions and influence enzymatic function. FTO catalyzes removal of methyl groups from RNA [23, 34], and its activity is sensitive to oxygen availability, with reduced catalytic efficiency reported under hypoxic or ischemic conditions, including myocardial ischemia [21, 39].

Through its m6A demethylase activity, FTO has been linked to a broad range of biological processes, including DNA damage responses, heart failure, obesity, regulation of dopaminergic signaling, and neural development and regeneration, and has been associated with disorders such as diabetes and Alzheimer’s disease [23, 40, 61]. Within the nervous system, FTO expression is dynamically regulated during learning. During memory formation, FTO levels transiently decrease, including at synaptic sites, and knockdown of Fto prior to behavioral training enhances contextual fear memory, consistent with a modulatory role for FTO in memory encoding [9].

ALKBH5 was identified subsequently as a second m6A demethylase. In contrast to FTO, which may proceed through multiple oxidative intermediates, ALKBH5 directly converts m6A to adenosine without detectable by-products [22]. In the mouse brain, *Alkbh5* is broadly expressed, with particularly high levels in the cerebellum and olfactory bulb in adulthood [45]. Immunostaining studies indicate that ALKBH5 is predominantly localized to the nucleus in neurons and cultured cell lines, where it participates in processes related to synaptic tagging that contribute to memory formation [41]. During brain development, ALKBH5 protein levels gradually decline, consistent with developmentally regulated control of m6A demethylation capacity [45].

### Reader proteins

The functional consequences of m6A deposition are largely determined by reader proteins that selectively bind methylated RNA and influence its post-transcriptional fate. The best-characterized m6A readers belong to the YTH domain family, including YTHDF1 and YTHDC1, with additional members such as YTHDF2 and YTHDF3 playing prominent roles in mRNA regulation [19, 53]. These proteins share a conserved m6A-binding module and preferentially recognize consensus RNA motifs containing the modification. Through interactions with distinct readers, m6A-marked transcripts are routed into coordinated regulatory pathways governing splicing, nuclear export, translation, decay, and subcellular localization.

Functional specialization among YTH family members enables context-dependent regulation of gene expression. Binding of YTHDF2 promotes mRNA degradation, thereby shaping transcript abundance and turnover [62], whereas YTHDF1 and YTHDF3 are associated with enhanced translation through recruitment of the translational initiation machinery [63]. In the hippocampus, YTHDF1 has been linked to learning and memory formation [46]. It supports activity-dependent translation of m6A-modified neuronal transcripts, and loss of YTHDF1 is associated with impaired synaptic plasticity and memory performance; these effects are reversible upon restoration of its expression. Together, these observations support the view that m6A-dependent reader engagement is associated with selective translational control during memory encoding.

Other YTH readers exert broader regulatory influences. YTHDC1 participates in multiple aspects of RNA metabolism, including alternative splicing, accelerated nuclear export, and selective RNA turnover [64]. YTHDC2 contributes to mRNA stability and is associated with modulation of translational efficiency. In contrast to YTHDF2-mediated decay, members of the IGF2BP family act as m6A readers that stabilize methylated transcripts and prolong their expression [53], highlighting how distinct readers are associated with opposing outcomes on shared RNA targets.

Beyond YTH domain proteins, additional m6A readers expand the functional diversity of this regulatory system. PRRC2A has been identified as an m6A reader associated with stabilization of transcripts required for oligodendrocyte progenitor differentiation and myelination, supporting lineage specification programs in the nervous system [57]. HNRNPC and HNRNPG, members of the heterogeneous nuclear ribonucleoprotein family, recognize m6A-modified RNA indirectly through an “m6A switch” mechanism. Rather than binding the modification itself, they sense methylation-induced changes in RNA secondary structure and influence downstream RNA processing. In the brain, these factors are associated with regulation of neuronal mRNA splicing, stability, and transport, contributing to synaptic plasticity and neurodevelopment, and their dysregulation has been linked to neurodegenerative conditions [58].

## Spatial dynamics of m6A in the brain

Compared with other tissues, m6A is markedly enriched in the nervous system, with particularly high abundance in the mammalian brain [65]. Through m6A-dependent regulatory mechanisms, this modification is associated with regulation of the expression and turnover of transcripts involved in neural development, shaping gene networks that govern cell fate decisions during embryonic and postnatal stages. Consistent with this association, disruption of m6A homeostasis has been linked to developmental abnormalities in the cerebral cortex and cerebellum [52]. Across development, global m6A levels in the brain increase progressively from embryogenesis to adulthood, and a substantial fraction of genes stably expressed in adult mice retain m6A marks [52].

At the cellular level, m6A is distributed across major brain cell populations. Transcriptome-wide analyses indicate higher m6A abundance in neurons than in glial cells [8, 10]. Subcellular mapping further reveals preferential enrichment of m6A-modified transcripts within axons and dendrites rather than neuronal somata [66], consistent with local RNA regulation. Synapse-associated transcripts are frequently modified by m6A in both human brain datasets and mouse synaptosome preparations [52, 67]. Beyond neurons, m6A is also detected in microglia and astrocytes [68] and has been linked to oligodendrocyte proliferation and lineage progression, influencing physiological functions and developmental fate decisions within the glial compartment [41].

In addition to cell-type differences, m6A distribution also varies across brain regions. The hippocampus shows activity- and age-dependent changes in m6A, with modified transcripts enriched in genes related to synaptic function and plasticity. Altered hippocampal m6A patterns have been reported in both mouse models and post-mortem human samples of neurodegenerative disease. The prefrontal cortex likewise exhibits activity- and stress-associated shifts in m6A profiles, reflecting its role in executive and emotional regulation.

Accumulating evidence indicates that cell-type-specific alterations in m6A regulation are associated with disease susceptibility. In astrocytes, changes in m6A have been linked to neuroinflammatory responses in conditions such as stroke and depression, while microglial m6A regulation has been associated with immune activation [69]. Dysregulation of m6A in oligodendrocytes has been associated with impaired myelination and cognitive decline [12]. Together, these observations underscore how the spatial organization of m6A across cell types and subcellular domains is associated with both physiological neural communication and vulnerability to neuropsychiatric and neurodegenerative disorders [13, 70].

## m6A in brain development

**Fig. 1:**
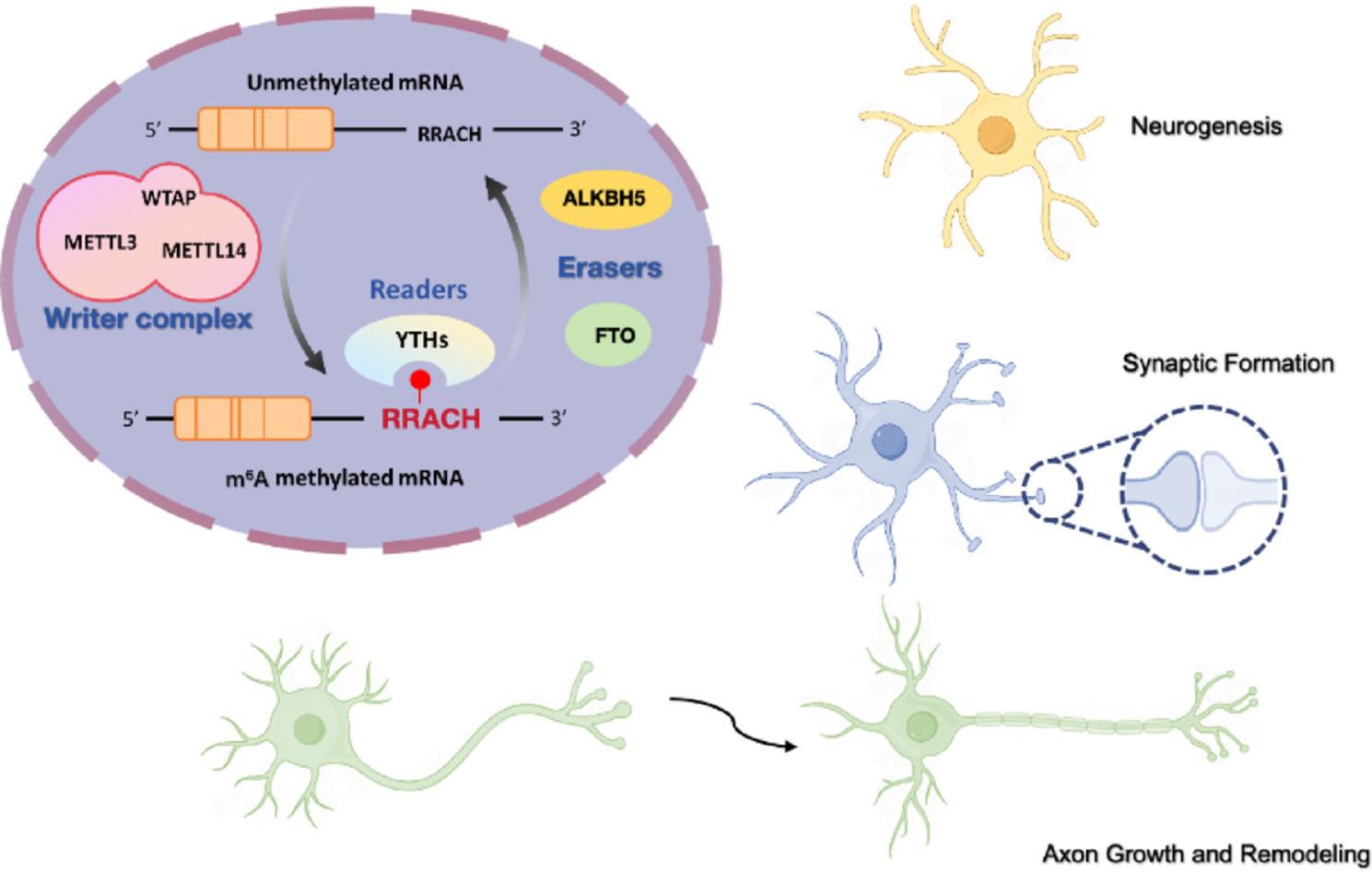
m6A RNA methylation in brain development. This schematic summarizes the role of m6A RNA methylation in neural stem cell proliferation, lineage specification, synaptogenesis, and axon growth. The m6A methyltransferase complex (writers METTL3, METTL14, and WTAP), demethylases (FTO and ALKBH5), and reader proteins (e.g., YTHDF family members) coordinate RNA splicing, stability, export, and translation. Through regulated deposition and interpretation of m6A marks, these components control the timing of neurogenesis and the assembly of functional neural circuits during early brain development. Arrows indicate reported increases or decreases in regulatory activity. m6A, N6-methyladenosine; METTL3, methyltransferase-like 3; METTL14, methyltransferase-like 14; WTAP, Wilms tumor 1 associated protein; FTO, fat mass and obesity-associated protein; ALKBH5, alkB homolog 5, RNA demethylase; YTHs, YTH family proteins; RRACH, m6A consensus motif (R = A/G, H = A/C/U)

m6A levels are high in the brain during embryonic and early postnatal stages and have been associated with multiple developmental programs [66]. The enzymes that regulate m6A, including methyltransferases, demethylases, and reader proteins, are broadly distributed across major brain regions. Evidence indicates that m6A is associated with regulation of neural stem cell proliferation and differentiation, synapse formation, and circuit assembly through modulation of RNA splicing, stability, and translation. m6A has also been linked to interactions with transcription factors and signaling pathways that shape gene expression programs relevant to neurodevelopment. Collectively, these regulatory processes are associated with neurogenesis, synaptogenesis, and axon growth and remodeling. An overview of these actions is presented in Fig. 1.

### m6A in neurogenesis

Adult hippocampal neurogenesis contributes to learning, memory, and emotional regulation, and its disruption has been linked to multiple brain disorders. Although neurogenesis declines after birth, m6A-mediated regulation remains important for maintaining neural stem cell renewal and differentiation throughout life [9, 71]. Recent studies indicate that the METTL3–METTL14 methyltransferase complex is associated with control of the timing of cortical neurogenesis [52]. METTL3 is highly expressed during early development and has been linked to neural differentiation, in part through regulation of the histone methyltransferase Ezh2 [71]. Genetic deletion of METTL3 or METTL14 in neural progenitor cells reduces proliferative capacity, prolongs the cell cycle, and delays postnatal neurogenesis, leading to a shift toward glial fate and impaired maturation of newborn neurons. Partial rescue of these phenotypes by restoring Ezh2 expression supports an association between m6A-dependent RNA regulation and chromatin-based control of neurogenic programs [9, 71].

m6A demethylation is also associated with postnatal neurogenesis. Mice lacking FTO, the first identified m6A demethylase, exhibit reduced neural stem cell pools and impaired hippocampal neurogenesis, accompanied by learning and memory deficits and decreased brain volume [35, 72, 73]. Mechanistic analyses indicate that loss of FTO is associated with increased m6A levels on transcripts involved in BDNF signaling, promoting their degradation and reducing neurogenic capacity [35, 50]. In parallel, m6A reader proteins contribute to regulation of neurogenesis by shaping transcript stability. YTHDF2 promotes decay of neural development–related mRNAs and is associated with regulation of stem cell behavior. Deletion of *Ythdf2* in early postnatal hippocampal neural stem cells disrupts neurogenesis and activates TGF-β signaling pathways linked to stem cell quiescence [74]. Similarly, embryonic neural stem cells lacking *Ythdf2* show reduced proliferation and differentiation, abnormal neurite outgrowth, and increased sensitivity to oxidative stress [75].

Together, these findings support the view that balanced m6A methylation, demethylation, and reader-mediated interpretation are associated with proper neural stem cell function during both embryonic brain development and adult hippocampal neurogenesis.

### m6A in synaptic formation

During brain development, coordinated neuronal migration is essential for establishing cortical architecture. Neurons generated in the ventricular zone migrate along radial or tangential routes to reach their final positions in a tightly regulated temporal sequence. Emerging evidence indicates that m6A is associated with this process through its influence on neuronal mRNA localization, enabling spatially restricted gene expression programs relevant to neuronal positioning and connectivity [76]. In parallel, m6A has been linked to synapse formation through regulation of translation of transcripts encoding synaptic proteins. High-throughput analyses suggest that synaptic plasticity engages post-transcriptional regulatory pathways, including phosphorylation-dependent signaling, and that m6A-modified transcripts are integrated into these programs [77].

m6A-dependent control of local protein synthesis has been demonstrated in motor neurons, where this modification regulates translation within axons [78]. Comparable mechanisms are thought to operate at dendritic synapses, where m6A is associated with local translation of proteins required for synaptic function and maturation [41]. Disruption of this regulatory layer has been associated with aging-related changes and has been implicated in neurodegenerative disorders, including Alzheimer’s disease [52].

In hippocampal neurons, altered m6A levels have been linked to disrupted translation of mRNAs involved in dendritic architecture, resulting in impaired synaptic transmission and abnormal dendritic morphology [79]. The m6A demethylase FTO is localized near synapses, and its expression decreases during learning, consistent with association with experience-dependent modulation of synaptic function [36]. The reader protein YTHDF1 is enriched in dendritic spines and is upregulated following fear conditioning; loss of YTHDF1 has been associated with impaired long-term potentiation, reduced spine density, and disrupted hippocampus-dependent learning, supporting a role for YTHDF1-mediated translation in synaptic protein synthesis [46]. Additional reader proteins may also be associated with synaptic function through regulation of stability, transport, or compartmentalization of synaptic mRNAs [79].

### *m6A in axon growth and remodeling*

Transport of mRNAs to distal neuronal compartments, including axons and dendrites, enables localized protein synthesis that is essential for neural development and plasticity. This spatially restricted translational control supports axon elongation, guidance, and synaptic remodeling. m6A has been linked to regulation of axonal mRNA localization and translation [78]. Loss of METTL3 alters the neuritic distribution of numerous transcripts, indicating that m6A is associated with proper positioning of RNAs required for axonal structure and growth. In parallel, the m6A demethylase FTO is enriched in axons and modulates local transcript stability. Within axonal compartments, FTO deficiency increases m6A levels on GAP43 mRNA, reduces its translation, and is associated with impaired axon outgrowth [72].

m6A reader proteins further refine axonal patterning by directing selective translation of guidance-related transcripts. YTHDF1 enhances translation of Robo3.1, a guidance molecule required for midline crossing during development, and has been linked to axon guidance decisions [47, 76]. Components of the Wnt5a signaling pathway, which contributes to axonal patterning, are also regulated by YTHDF1 and YTHDF2 [48]. m6A-dependent translation of adenomatous polyposis coli (APC) further links epitranscriptomic regulation to axon development. APC mRNA carries m6A modifications that promote its translation via YTHDF proteins; reduced METTL14 or YTHDF1 impairs APC expression, disrupts local β-actin translation in axons, and compromises axon development [80]. Together, these observations support an association between m6A reader activity and coordination of axon navigation and growth through localized translational control.

Beyond development, m6A is also engaged in neural repair processes. Following axonal injury, m6A levels increase in affected neurons and are associated with enhanced translation of regeneration-associated transcripts [67]. In dorsal root ganglion neurons and retinal ganglion cells, deletion of METTL14 or YTHDF1 compromises this response by reducing protein synthesis required for effective regeneration [81]. Collectively, these findings indicate that balanced m6A methylation and reader-mediated interpretation are associated with both developmental axon growth and axonal repair in the peripheral and central nervous systems.

## m6A in brain aging

The aging brain is exposed to accumulating cellular stressors, including misfolded protein burden, reduced RNA synthesis, mitochondrial dysfunction, and increased DNA damage and oxidative stress [82]. Aging is a major risk factor for cognitive decline and neurodegenerative disease, and molecular pathways that support synaptic plasticity and circuit stability earlier in life may become sources of vulnerability with advancing age. As a pervasive regulator of RNA metabolism, m6A has been linked to these age-associated processes.

m6A-modified transcripts are enriched in pathways critical for synaptic function and neuronal signaling, and both their expression levels and methylation patterns show age-dependent changes that vary across neuronal and glial cell types [83]. Single-cell m6A transcriptomic profiling of young and aged mouse brains has identified hundreds of transcripts with age-related differences in methylation [84]. Consistent with these findings, MeRIP-seq analysis of hippocampal RNA from young (3-month-old) and aged (20-month-old) mice revealed more than 8,000 m6A peaks in young animals and approximately 7500 in aged animals, mapping to over 4,500 protein-coding genes in both groups. In both age cohorts, m6A peaks were preferentially enriched within exons and 3′ untranslated regions near stop codons, and the conserved GGAC motif remained prominent [85].

At the global level, m6A abundance increases with age in the mouse brain and in models expressing human Aβ42 [83]. Functional studies further indicate that m6A regulation exhibits cell-type-specific associations during aging. Neuronal knockdown of *Mettl3* shortens lifespan and elevates markers of DNA damage while increasing translation efficiency of m6A-tagged transcripts, whereas glial-specific *Mettl3* knockdown extends lifespan but reduces translation efficiency, highlighting divergent roles for m6A regulation across cellular compartments [86]. Region-specific analyses of mouse and human brain tissue have additionally identified reduced m6A modification on synaptic genes such as *CAMKII* and *GluA1* in aged animals and in patients with Alzheimer’s disease [86].

Together, these findings support the view that m6A is associated with transcriptomic adaptation across the lifespan, influencing both physiological aging and disease-related trajectories. Age-related disruption of m6A regulation has been linked to compromised synaptic resilience and increased susceptibility to cognitive decline and neurodegenerative processes.

## m6A as a mediator of experience-dependent plasticity

As the brain matures, experience becomes an increasingly prominent influence on neural structure and function. The nervous system retains the capacity for plasticity across the lifespan, enabling structural and functional remodeling in response to environmental input, as originally demonstrated by enriched-environment studies [87]. Synaptic plasticity provides the cellular substrate for learning and memory and depends on activity-dependent transcription and protein synthesis that stabilize enduring changes in synaptic strength [1, 50, 88]. Experience-dependent plasticity encompasses reorganization of neural circuits driven by sensory stimulation, learning, behavioral training, and injury. This process operates throughout life and is modulated by factors such as age, sex, and genetic background. Converging evidence from neuroimaging and experimental models indicates that synaptic and structural remodeling can persist well into adulthood.

Within this framework, m6A has been linked to post-transcriptional mechanisms that couple neuronal activity to molecular adaptation. In mature neurons, m6A is associated with regulation of mRNA transport, stability, and local translation, with particular relevance at synaptic sites [41]. Following behavioral training, m6A dynamically modulates transcripts encoding immediate early genes, and both m6A-modified RNAs and components of the m6A machinery become enriched at synapses [73]. Although aging is associated with increased vulnerability of m6A regulatory programs, their dynamic regulation also supports adaptive responses to experience across the lifespan. During early development, circuit assembly is largely guided by genetic programs; however, functional studies indicate that disruption of m6A regulation at later stages is associated with impaired synaptic transmission and plasticity, as well as learning and memory deficits in animal models [86]. Consistent with activity-dependent regulation, behavioral stimulation in mice increases m6A accumulation in the prefrontal cortex, supporting an association between m6A and experience-driven gene regulation during memory processing [89]. Despite these advances, the molecular mechanisms through which m6A links experience to persistent circuit change remain incompletely defined.

**Fig. 2:**
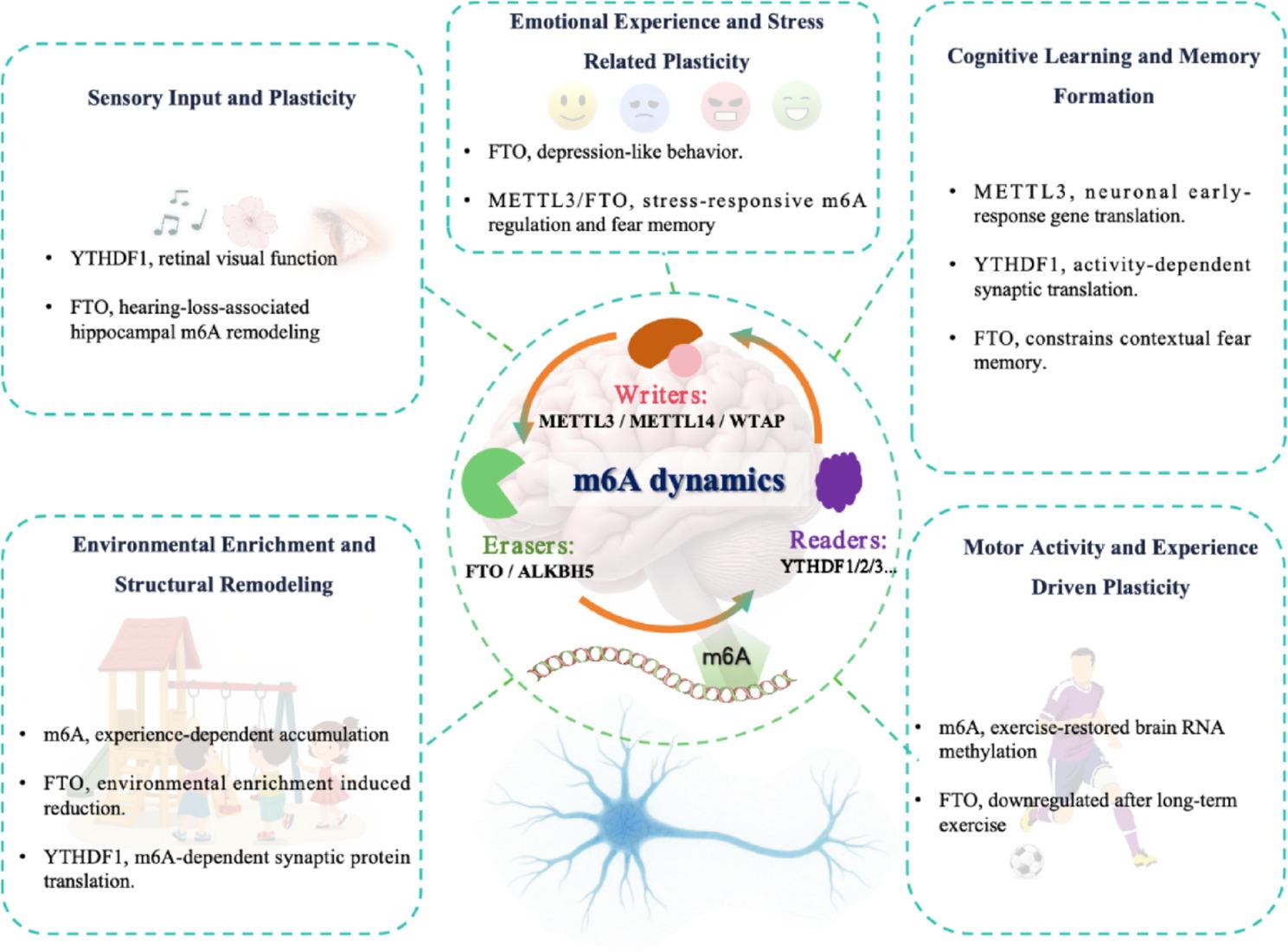
m6A RNA methylation and experience-dependent neural plasticity. This schematic illustrates how dynamic m6A regulation participates in distinct domains of experience-dependent neural plasticity, including sensory input, emotional experience, cognitive learning and memory formation, environmental enrichment, and motor activity. The coordinated actions of m6A “writers” (e.g., METTL3/METTL14), “erasers” (FTO, ALKBH5), and “readers” (e.g., YTHDF family proteins) regulate transcript stability, localization, and activity-dependent translation in mature neurons. Representative molecular components are included to illustrate experimentally supported links between m6A regulation and domain-specific forms of activity-dependent synaptic remodeling. In hippocampal and prefrontal cortical circuits, activity-induced changes in m6A deposition influence immediate-early gene expression and synaptic protein synthesis, thereby shaping synaptic remodeling and adaptive behavioral responses. By integrating molecular regulation with circuit-level plasticity, m6A acts as a dynamic interface between experience and neuronal connectivity. m6A, N6-methyladenosine; METTL3, methyltransferase-like 3; METTL14, methyltransferase-like 14; FTO, fat mass and obesity-associated protein; ALKBH5, AlkB homolog 5; YTHDF1, YTH N6-methyladenosine protein 1; YTHDF2, YTH N6-methyladenosine RNA binding protein 2; YTHDF3, YTH N6-methyladenosine RNA binding protein 3; RRACH, m6A consensus motif (R = G/A; H = A/C/U)

In the following sections, we examine how m6A participates in experience-dependent plasticity across distinct behavioral and environmental contexts. As outlined in Fig. 2, we focus on five interrelated domains through which experience shapes neural organization: sensory input, cognitive learning, environmental enrichment, emotional influences, and motor activity. Together, these domains provide a framework for understanding how m6A-mediated RNA regulation is associated with brain plasticity across the lifespan.

### Sensory input and plasticity

During early childhood, particularly within the first three years of life, the brain undergoes rapid structural and functional maturation. Sensory input during this period is critical for normal circuit formation and the establishment of plasticity mechanisms [90, 91]. Disruption of sensory experience during this window can therefore have lasting consequences. For example, deprivation of visual input in infancy impairs development of the visual cortex and has been linked to long-term deficits in other sensorimotor functions [92]. Experimental studies in young cats further show that even brief periods of visual deprivation induce measurable structural alterations in the brain, whereas prolonged deprivation is associated with irreversible injury [93]. Recent molecular studies suggest that m6A-dependent regulation is associated with sensory system function and vulnerability. Single-cell analyses have identified the m6A reader YTHDF1 as required for normal retinal function, where it regulates translation of TULP1 and DHX38, two factors essential for visual processing. These observations support an association between m6A-mediated translational control and sensory-related neurodegeneration [51]. Auditory input is similarly important for circuit maturation. Early-life hearing loss has been shown to impair hippocampal synaptic plasticity and to be associated with deficits in learning and memory [94, 95]. In mouse models of developmental hearing loss, hippocampal m6A landscapes are altered in adulthood, characterized by increased FTO expression and multiple differentially methylated regions, suggesting that m6A regulation may contribute to long-term neural responses to auditory deprivation [96].

Taken together, current evidence supports a role for m6A in the brain’s adaptation to sensory experience during development. The precise mechanisms remain to be clarified, and further studies are needed to determine how m6A contributes to sensory-driven plasticity and whether it can be targeted to support neural development in children with early sensory deficits.

### Cognitive learning and memory formation

Learning experiences during infancy and early childhood contribute to brain maturation by shaping both cortical structure and function. Interaction with the environment, exposure to language, and engagement in formal or informal education can remodel specific cortical regions. For example, repeated musical training in children alters the organization of motor and auditory cortices, illustrating the capacity of the developing brain to adapt to cognitive experience [97]. At the cellular level, memory formation depends on synaptic plasticity. Transient forms of plasticity are often supported by local protein synthesis at synapses, whereas stable long-term memory requires coordinated nuclear transcription. Integration of these processes supports memory consolidation and the persistence of learning-related circuit changes [1]. Central to this coordination is activity-dependent protein synthesis, which reinforces synaptic modifications associated with learning.

Within this framework, m6A has been linked to post-transcriptional regulation that coordinates neuronal responses to synaptic activity. Disruption of m6A regulation has been associated with impairments in learning and memory. Loss of METTL3 attenuates rapid transcriptional activation of immediate early genes required for memory formation [89]. METTL14 is similarly associated with maintenance of neuronal excitability and support of memory consolidation. In contrast, hippocampal deletion of FTO enhances contextual fear memory, consistent with a modulatory role for m6A demethylation in emotional learning [89]. At the level of translational control, the m6A reader YTHDF1 promotes activity-dependent translation of synaptic transcripts, and its deletion is associated with impaired hippocampal synaptic transmission and deficits in memory performance [63].

In summary, m6A acting through METTL3, METTL14, FTO, and YTHDF1 influences learning-induced gene expression and synaptic function. These regulatory pathways are essential for memory encoding.

### Environmental enrichment and structural remodeling

Environmental stimulation is a well-established driver of brain plasticity. Animals raised in enriched environments exhibit increased dendritic branching and higher synaptic density in cortical regions compared with those housed under standard conditions [98]. In rodents, environmental enrichment is also associated with changes in brain weight, cortical thickness, acetylcholine levels, and dendritic architecture, indicating that environmental experience reshapes both the structural and chemical organization of the brain [99]. These effects reflect interactions between environmental input and intrinsic genetic programs, with epigenetic mechanisms, including m6A RNA methylation, implicated in this integration. Through activity-dependent regulation of gene expression, m6A has been linked to experience-associated modulation of neurogenesis and synaptic plasticity [89].

In enriched environments, elevated neuronal activity increases the demand for protein synthesis required to support memory formation. In hippocampal neurons, the m6A reader YTHDF1 facilitates activity-dependent translation of specific target transcripts, and loss of YTHDF1 is associated with impaired synaptic transmission and long-term potentiation, linking m6A reader function to molecular processes that support memory-related plasticity [46]. Together, these observations indicate that m6A contributes to structural and functional remodeling of the brain in response to enriching environmental conditions.

Future studies should clarify how region-specific m6A dynamics coordinate long-term structural adaptation under sustained environmental stimulation.

### Emotional experience and stress-related plasticity

Emotional experiences shape neural circuit function across development. Chronic or adverse stress has been linked to impaired neural maturation and increased vulnerability to psychiatric disorders. For example, chronic peer victimization during adolescence has been associated with altered trajectories of brain development, suggesting that early psychosocial stress can produce lasting neurobiological changes [100]. Such findings underscore the sensitivity of developing neural circuits to stress exposure during critical periods.

Stress is a potent modulator of neural plasticity. Elevated glucocorticoid levels have been associated with impaired memory function [101], and repeated stress exposure has been linked to remodeling of dendritic architecture in the hippocampus. Early-life stress can further sensitize stress-responsive systems later in life, often accompanied by reduced neurogenesis. Together, these observations highlight how emotional and stress-related experiences converge on neural circuits involved in learning, memory, and affective regulation [44].

Reduced FTO expression has been reported in the hippocampus of patients with major depressive disorder and in corresponding animal models. Experimental suppression of FTO is associated with depression-like behaviors, whereas FTO overexpression reverses these effects, supporting an association between m6A regulation and mood-related pathology. Alterations in fear memory processing further link m6A to stress-related plasticity. Fear memory generalization, a feature of conditions such as post-traumatic stress disorder, has been associated with altered m6A regulation. Higher global m6A levels correlate with improved threat discrimination, and hippocampal METTL3 expression has been linked to accurate fear memory retrieval, consistent with m6A-associated modulation of glutamatergic signaling during emotional learning [42].

Together, these findings support a role for m6A in shaping neural responses to emotional and stress-related experiences. Further work is needed to define how cell-type– and region-specific m6A dynamics contribute to individual differences in stress resilience and vulnerability to mood disorders.

### Motor activity and experience-driven plasticity

Physical activity and motor training exert pronounced effects on brain structure and function. In animal models, exposure to motorically challenging environments is associated with enlargement of the motor cortex, indicating that repetitive motor activity enhances synaptic connectivity and neural circuit efficiency [102]. Regular treadmill running further promotes stabilization of newly formed synapses by increasing dendritic spine density [102]. Across aging and disease models, exercise has been linked to improved cognitive performance, likely through reinforcement of neuroplastic mechanisms that support motor learning and functional recovery [103].

At the molecular level, motor experience has been associated with changes in m6A RNA methylation within neurons. Exercise increases m6A levels on transcripts related to synaptic signaling, consistent with elevated demands for activity-dependent gene regulation. In contrast, reduced m6A methylation in hippocampal neurons has been linked to immature dendritic spine morphology, weakened excitatory synaptic transmission, and decreased levels of postsynaptic density protein 95, collectively compromising synaptic function [104]. Motor activity also influences expression of enzymes involved in RNA methylation. Long-term physical training reduces FTO expression in both the hippocampus and hypothalamus [105], suggesting an association between m6A demethylation and adaptive brain remodeling in response to motor activity.

Transport and local translation of mRNAs at synapses are essential for maintaining structural plasticity. Disruption of m6A regulation has been linked to interference with dendritic mRNA translation and local protein synthesis, thereby compromising synaptic efficiency. In cultured hippocampal neurons, aberrant m6A levels disrupt translation of mRNAs associated with dendritic localization, resulting in impaired synaptic transmission and abnormal dendritic morphology [48].

In summary, motor activity dynamically modulates m6A methylation, contributing to structural remodeling of the nervous system. These changes support synaptic plasticity and may underlie the beneficial effects of physical training on learning and motor recovery.

## m6A dysregulation in neurodegenerative and cognitive disorders

Neural plasticity is essential for learning, memory, and adaptive brain function. An increasing body of evidence has linked alterations in m6A regulation to several neurological disorders, including Alzheimer’s disease, Parkinson’s disease, and Huntington’s disease. Beyond these classical neurodegenerative conditions, m6A dysregulation has also been implicated in stroke-related cognitive decline and in neurotoxicity associated with environmental or metal exposures. Across these contexts, altered m6A regulation is associated with changes in post-transcriptional gene control, with potential consequences for neuronal survival, synaptic plasticity, and inflammatory processes.

**Fig. 3:**
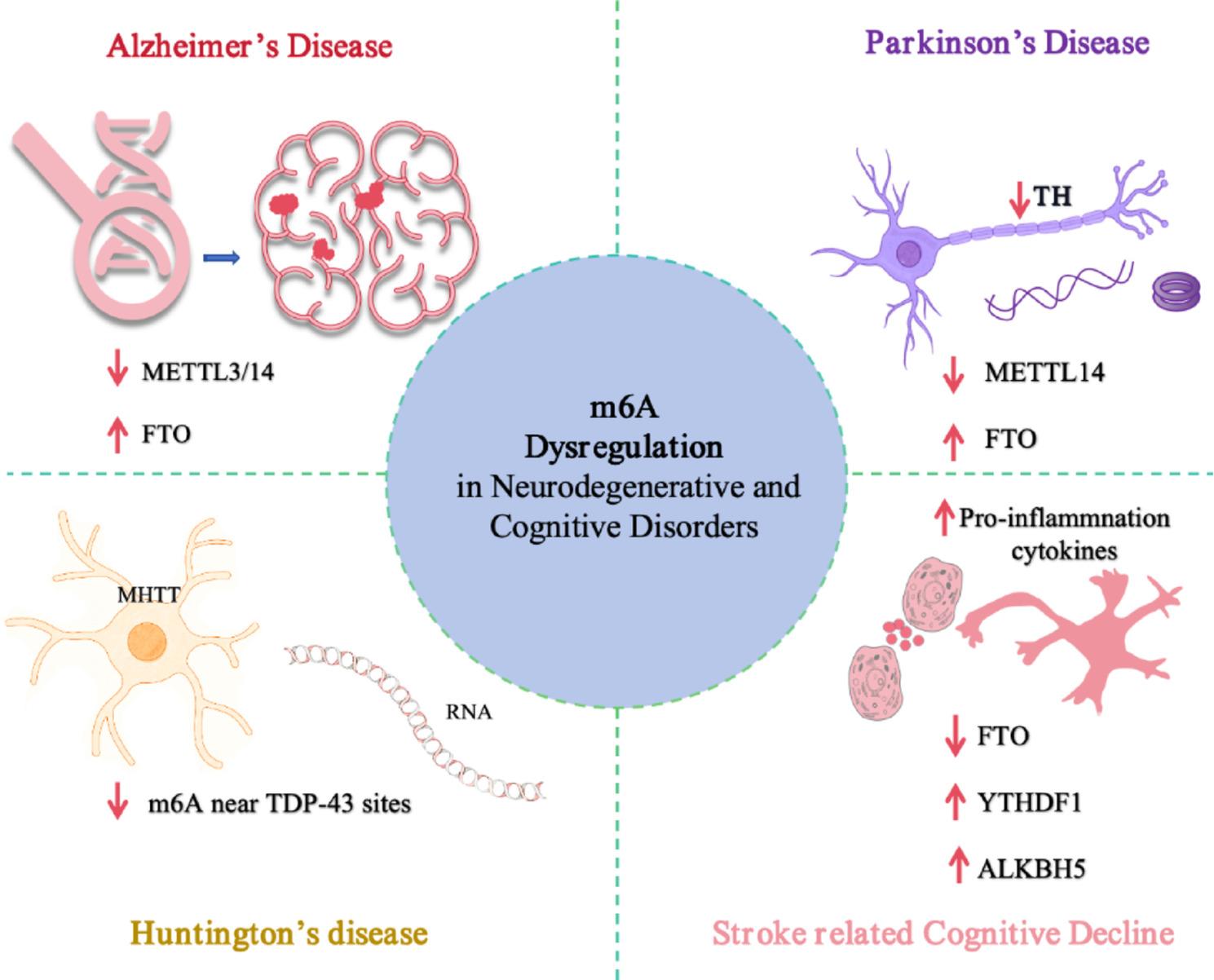
m6A dysregulation across major neurodegenerative and cognitive disorders. This schematic summarizes disease-associated alterations in m6A regulatory components across Alzheimer’s disease (AD), Parkinson’s disease (PD), Huntington’s disease (HD), and stroke-related cognitive decline. In AD, reduced METTL3/METTL14 and elevated FTO are associated with Tau pathology and mitochondrial dysfunction. In PD, decreased METTL14 and increased FTO disrupt dopaminergic transcript regulation, including tyrosine hydroxylase (TH). In HD, mutant huntingtin (mHTT) accumulation coincides with altered m6A deposition near TDP-43 binding sites, a change associated with RNA processing abnormalities observed in striatal neurons. In ischemic injury and stroke-related cognitive decline, decreased FTO, increased global m6A, and altered YTHDF1 and ALKBH5 expression are linked to inflammatory signaling and neuronal apoptosis. Arrows indicate direction of reported change. m6A, N6-methyladenosine; METTL3/14, methyltransferase-like 3/14; FTO, fat mass and obesity-associated protein; YTHDF1, YTH N6-methyladenosine RNA binding protein 1; ALKBH5, AlkB homolog 5; TH, tyrosine hydroxylase; mHTT, mutant huntingtin; TAR DNA-binding protein 43

Because these disorders are commonly characterized by progressive cognitive impairment and functional deterioration, defining the involvement of m6A in disease-associated molecular pathways may help clarify underlying mechanisms. A more precise understanding of how m6A regulation is altered in pathological states could also inform future efforts aimed at improving disease characterization and intervention strategies. Figure 3 provides a mechanistic overview of m6A dysregulation across major neurodegenerative disorders.

### m6A in Alzheimer’s disease

Alzheimer’s disease (AD) is a progressive neurodegenerative disorder characterized by cognitive decline and functional deterioration [106]. Although the pathogenic cascade remains incompletely defined, epigenetic dysregulation is increasingly recognized as a contributing factor, and m6A RNA methylation has been linked to synaptic regulation and early disease-related processes [20]. Postmortem analyses of the AD cortex reveal abnormal m6A patterns, with comparable alterations reported in animal models [106]. Among m6A regulators, reduced METTL3 expression has been observed in the hippocampus and cortex of patients with AD and has been associated with increased Tau phosphorylation, a central feature of AD pathology [25, 107]. Decreased levels of METTL3 and METTL14 have also been reported in individuals with mild cognitive impairment, suggesting that m6A dysregulation may occur prior to overt clinical diagnosis [107].

In AD model mice, METTL3 downregulation has been associated with synaptic loss, neuronal death, and memory impairment [25, 107]. Conversely, experimental upregulation of METTL3 has been reported to improve mitochondrial function and cognitive performance, potentially through m6A-dependent enhancement of MFN2 expression [108]. Region-specific alterations in synapse-related m6A modifications have also been described and may reflect differences in the expression or activity of m6A reader proteins, including members of the YTH domain family [109].

m6A demethylases have likewise been linked to AD-related pathology. FTO expression is increased in AD model brains and has been associated with exacerbation of disease-related cognitive deficits. In contrast, reduction of FTO levels has been linked to decreased Tau phosphorylation while exerting minimal effects on amyloid deposition [25]. Transcriptomic analyses of AD brain tissue further reveal upregulation of IGF2BP2, an m6A reader involved in mRNA stabilization, suggesting a potential contribution to disease progression [54].

In summary, multiple components of the m6A regulatory machinery, including METTL3, METTL14, FTO, and IGF2BP2, have been linked to AD-associated molecular and cellular changes. However, their precise roles in synaptic integrity, neuronal survival, and cognitive dysfunction remain to be fully defined.

### m6A in Parkinson’s disease

Parkinson’s disease (PD) is an age-related neurodegenerative disorder characterized by progressive loss of dopaminergic neurons in the substantia nigra, reduced dopamine production, and motor symptoms including tremor, rigidity, and bradykinesia [110]. Although the etiological mechanisms underlying PD remain incompletely understood, epigenetic regulation has received increasing attention, and m6A RNA methylation has been linked to disease-associated molecular alterations [111].

Experimental evidence indicates that m6A dysregulation is associated with changes in dopaminergic signaling pathways. In rat models, FTO deficiency has been linked to elevated m6A levels and reduced translation of transcripts involved in dopaminergic function [89]. In contrast, in a 6-hydroxydopamine–induced PD model, global m6A levels are reduced while FTO expression is increased relative to controls [79]. In vitro studies further show that FTO overexpression is associated with increased neuronal vulnerability to injury, consistent with stress-related pro-apoptotic effects, whereas FTO knockout has been linked to altered reward-learning behavior, a functional domain frequently affected in PD [26].

m6A writer enzymes have also been implicated in PD-related pathology. Selective deletion of METTL14 in the substantia nigra reduces expression of tyrosine hydroxylase, the rate-limiting enzyme in dopamine synthesis, and this reduction has been associated with motor impairment in experimental animals [27]. Together, these observations support an association between m6A-dependent regulation and alterations in dopaminergic signaling, synaptic plasticity, and neuronal survival in PD.

### 7.3 m6A in Huntington’s disease (HD)

Huntington’s disease (HD) is a progressive autosomal dominant neurodegenerative disorder caused by expansion of CAG repeats in the HTT gene. This mutation produces mutant huntingtin (mHTT) and is associated with selective loss of striatal GABAergic neurons, cortical atrophy, and progressive motor and cognitive impairment [112]. In addition to protein toxicity, the expanded repeat promotes somatic instability and disrupts RNA metabolism, including aberrant splicing events such as mHTT exon 1 transcripts and repeat-associated non-AUG (RAN) translation, processes that have been linked to exacerbation of disease pathology [113].

Disruption of RNA processing is increasingly recognized in HD. One proposed mechanism involves mislocalization of the RNA-binding protein TDP-43, a factor more commonly implicated in amyotrophic lateral sclerosis and frontotemporal dementia. In HD brain tissue, TDP-43 shows reduced binding to RNA targets involved in neuronal function, accompanied by decreased m6A modification near TDP-43–associated regions in the striatum, suggesting an interaction between altered RNA methylation and impaired splicing regulation [113]. Consistent with this observation, changes in m6A distribution have been associated with abnormal gene-expression patterns in striatal neurons. The presence of TDP-43 pathology in HD further supports the involvement of shared disturbances in RNA metabolism across neurodegenerative disorders.

In line with this view, m6A dysregulation has been reported in experimental models of both HD and ALS [114, 115]. Additional studies are needed to clarify how m6A-dependent RNA regulation is altered during disease progression and to assess its relevance to HD-associated molecular pathology.

### m6A in stroke-related cognitive decline

Post-stroke cognitive decline commonly affects memory, learning, and executive function and is frequently accompanied by motor and language impairments [116]. Secondary brain injury after stroke involves a combination of excitotoxicity, cerebral edema, oxidative stress, and inflammatory responses [116]. An increasing body of evidence has linked m6A RNA methylation to these pathological processes. In experimental models of cerebral ischemia, expression of the m6A demethylase FTO is reduced in brain tissue [23, 40]. This reduction is associated with increased global m6A levels and aggravated neuronal injury, whereas restoration of FTO expression has been reported to attenuate ischemic damage.

Altered activity of m6A reader proteins has also been observed following stroke. The m6A reader YTHDF1 is upregulated in the post-stroke brain and has been associated with inflammatory and apoptotic signaling through recognition of highly methylated transcripts involved in these pathways [117]. In neonatal hypoxic–ischemic injury, changes in m6A regulation have been linked to microglial activation and enhanced neuroinflammatory responses [118]. Additionally, the m6A demethylase ALKBH5 has been implicated in stroke-related neuronal apoptosis, in part through pathways involving endoplasmic reticulum stress and inflammation [119].

Collectively, these observations indicate that dysregulation of m6A regulators is closely associated with secondary brain injury and cognitive impairment following stroke.

### m6A in amyotrophic lateral sclerosis (ALS) and frontotemporal dementia (FTD)

Amyotrophic lateral sclerosis (ALS) and frontotemporal dementia (FTD) share overlapping genetic and pathological features despite distinct clinical manifestations. ALS primarily affects upper and lower motor neurons, whereas FTD is characterized by progressive deficits in behavior, language, and executive function. Both disorders commonly display abnormalities in RNA-binding proteins, including TDP-43, FUS, ATXN2, MATR3, and members of the heterogeneous nuclear ribonucleoprotein family. A recurrent genetic lesion in both ALS and FTD is the GGGGCC hexanucleotide repeat expansion in C9ORF72, which promotes the formation of RNA foci and the production of dipeptide repeat proteins. These species disrupt RNA-binding protein function and RNA metabolism and have been associated with neuronal dysfunction and cell death.

Accordingly, disruption of RNA processing—including splicing, transport, and RNA stability—occupies a central position in ALS and FTD pathogenesis. Accumulating evidence indicates that m6A RNA methylation intersects with these RNA regulatory pathways, although its precise contribution to disease mechanisms in ALS and FTD remains under active investigation [120].

### m6A in metal toxicity-related cognitive impairment

Emerging evidence has linked m6A RNA methylation to metal-induced cognitive dysfunction, in part through mechanisms involving oxidative stress and epigenetic regulation. Aluminum exposure suppresses expression of the m6A demethylase FTO and increases global m6A levels in hippocampal neurons, changes that are associated with aberrant methylation of BDNF mRNA and neuronal apoptosis [37]. In contrast, developmental aluminum exposure has been reported to reduce overall m6A levels and impair expression of key methyltransferases, alterations that correlate with long-term hippocampal damage [121].

Other environmental toxicants similarly perturb m6A regulatory pathways. Arsenic exposure decreases FTO expression through SUMOylation, leading to elevated m6A levels and increased oxidative injury mediated by IGF2BP3-associated mechanisms [55]. In dopaminergic neurons, arsenic-induced m6A accumulation has been linked to impaired neurotransmission, an effect that can be partially reversed by restoration of FTO expression [122]. The mycotoxin deoxynivalenol also alters m6A regulation in hippocampal neurons by increasing m6A modification of *p21* mRNA, enhancing its stability via YTHDF1 and IGF2BP1, while concurrently reducing *TRIM21* mRNA stability through YTHDF2, changes associated with impaired neuronal proliferation [49].

Collectively, these observations support the view that dysregulation of m6A represents a shared molecular feature of metal- and toxin-related neurotoxicity.

## Translational perspectives and summary

**Fig. 4:**
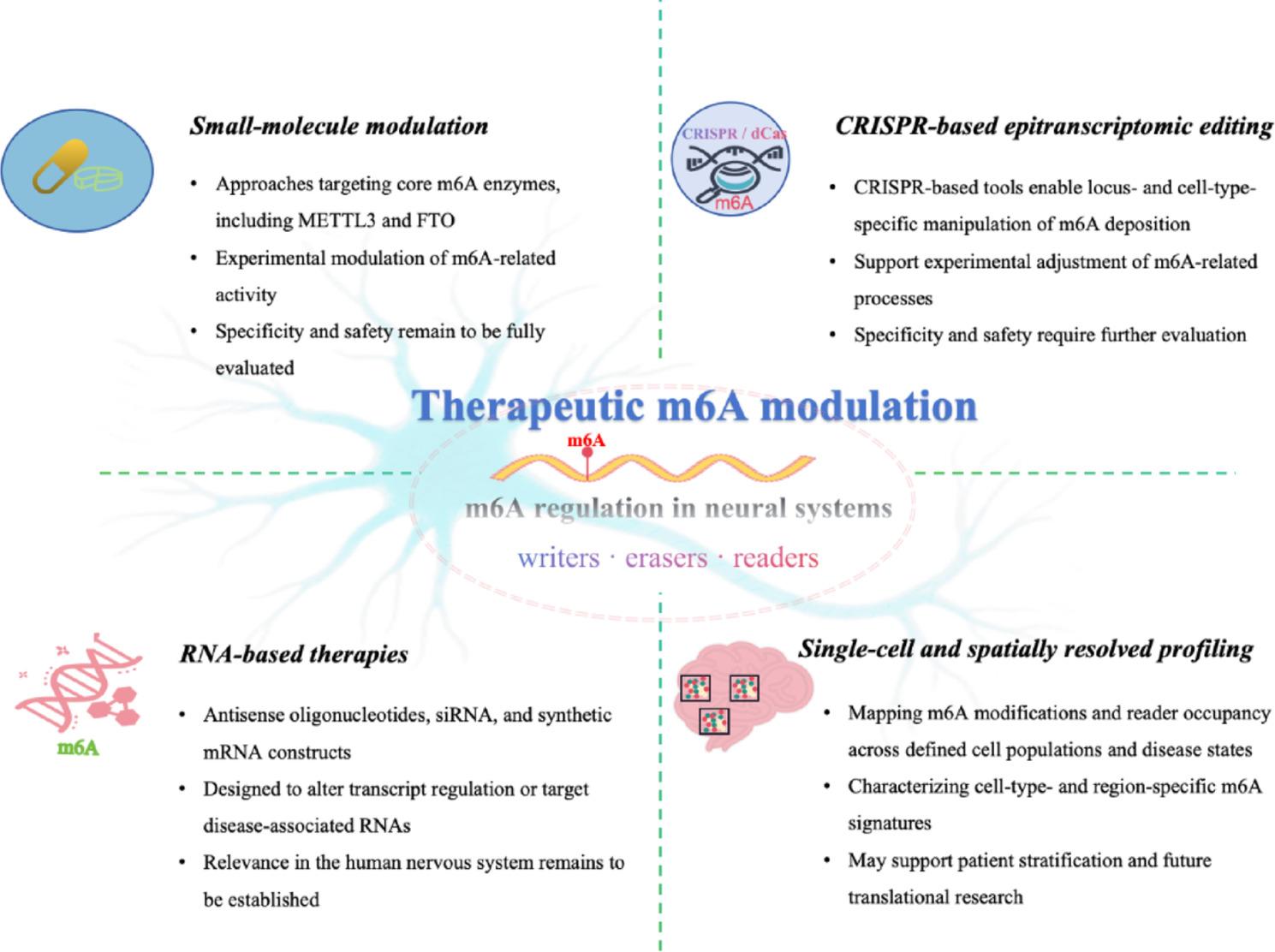
Translational perspectives on m6A regulation in neural systems. This schematic summarizes emerging approaches to modulate or interrogate m6A-related pathways in the nervous system. The central panel depicts m6A regulation as a dynamic and reversible layer of RNA control mediated by writers, erasers, and readers. Surrounding panels illustrate representative translational and analytical strategies discussed in the text, including small-molecule approaches targeting core m6A enzymes, RNA-based technologies such as antisense oligonucleotides, siRNA, and synthetic mRNA constructs, CRISPR-based tools enabling locus- and cell-type-specific manipulation of m6A deposition, and single-cell/spatially resolved methods for mapping m6A modifications and reader occupancy across defined cell populations and disease states. Together, these approaches support the experimental feasibility of adjusting m6A-related processes, although their specificity, safety, and relevance in the human nervous system remain to be fully established. m6A, N6-methyladenosine; METTL3, methyltransferase-like 3; FTO, fat mass and obesity-associated protein; ALKBH5, AlkB homolog 5

Over the past decade, studies of m6A RNA methylation have substantially advanced understanding of neural development, plasticity, and cognition. Accumulating evidence supports the view that dynamic m6A regulation is associated with experience-dependent gene expression, as well as with molecular changes observed during brain aging and neurodegeneration. Through coordinated control of RNA stability, translation, and localization, m6A provides a flexible regulatory layer linking neural activity to sustained adaptations in gene-expression programs. Despite this progress, key questions remain unresolved, including the site-specific functions of m6A across distinct cell types and brain regions and its interactions with other epigenetic mechanisms, such as DNA methylation, histone modification, and chromatin remodeling [123]. Figure 4 outlines potential therapeutic avenues spanning molecular, cellular, and circuit-level interventions.

Interest in translational applications of m6A-related pathways is increasing. Experimental studies have described multiple strategies capable of modulating m6A regulation, including approaches that influence the activity of core enzymes such as METTL3 and FTO [24], RNA-based technologies such as antisense oligonucleotides, siRNA, and synthetic mRNA constructs designed to alter transcript regulation or target disease-associated RNAs [124] and CRISPR-based tools enabling locus- and cell-type-specific manipulation of m6A deposition [125]. Together, these approaches demonstrate the feasibility of experimentally adjusting m6A-related processes, although their specificity, safety, and relevance in the human nervous system remain to be fully evaluated.

In parallel, advances in epitranscriptomic profiling are broadening translational perspectives. Single-cell and spatially resolved methods now permit mapping of m6A modifications and reader occupancy across defined cell populations and disease states [126]. These datasets support more refined characterization of cell-type- and region-specific m6A signatures and may aid in understanding molecular heterogeneity across neurological conditions. Integrating m6A-associated profiles with patient stratification, circuit-level analyses, and pharmacodynamic monitoring is likely to be important for future translational research.

In summary, current evidence supports the view that m6A represents a reversible layer of RNA regulation associated with neural experience, plasticity, and degeneration. Continued efforts to define its context-dependent functions and regulatory logic will be essential for advancing both mechanistic understanding and translational exploration.

## Data Availability

No datasets were generated or analysed during the current study.

## Abbreviations

m6A: N6-methyladenosine
AD: Alzheimer’s disease
PD: Parkinson’s disease
HD: Huntington’s disease
ALS: Amyotrophic lateral sclerosis
FTD: Frontotemporal dementia
ASO: Antisense oligonucleotide

## References

[1] Kirkwood A, Rioult MC, Bear MF. Experience-dependent modification of synaptic plasticity in visual cortex. Nature. 1996;381(6582):526–8. 8632826 10.1038/381526a0

[2] d’Aquin S, et al. Compartmentalized dendritic plasticity during associative learning. Science. 2022;376(6590):eabf7052. 35420958 10.1126/science.abf7052

[3] Holtmaat A, Svoboda K. Experience-dependent structural synaptic plasticity in the mammalian brain. Nat Rev Neurosci. 2009;10(9):647–58. 19693029 10.1038/nrn2699

[4] Zovkic IB, et al. Histone H2A.Z subunit exchange controls consolidation of recent and remote memory. Nature. 2014;515(7528):582–6. 25219850 10.1038/nature13707 PMC4768489

[5] Sweatt JD. The emerging field of neuroepigenetics. Neuron. 2013;80(3):624–32. 24183015 10.1016/j.neuron.2013.10.023 PMC3878295

[6] Hoernes TP, et al. Nucleotide modifications within bacterial messenger RNAs regulate their translation and are able to rewire the genetic code. Nucleic Acids Res. 2016;44(2):852–62. 26578598 10.1093/nar/gkv1182 PMC4737146

[7] Desrosiers R, Friderici K, Rottman F. Identification of methylated nucleosides in messenger RNA from Novikoff hepatoma cells. Proc Natl Acad Sci U S A. 1974;71(10):3971–5. 4372599 10.1073/pnas.71.10.3971 PMC434308

[8] Sweatt JD. Layered-up regulation in the developing brain. Nature. 2017;551(7681):448–9. 29168827 10.1038/d41586-017-07269-7 PMC6038706

[9] Yoon KJ, et al. Temporal control of mammalian cortical neurogenesis by m(6)A methylation. Cell. 2017;171(4):877–e88917. 28965759 10.1016/j.cell.2017.09.003 PMC5679435

[10] Livneh I, et al. The m(6)A epitranscriptome: transcriptome plasticity in brain development and function. Nat Rev Neurosci. 2020;21(1):36–51. 31804615 10.1038/s41583-019-0244-z

[11] Liu J, et al. Landscape and regulation of m(6)A and m(6)Am methylome across human and mouse tissues. Mol Cell. 2020;77(2):426–e4406. 31676230 10.1016/j.molcel.2019.09.032

[12] Yao M, et al. The impact of epigenetic and epitranscriptomic modifications on Alzheimer’s disease: the mysteries of DNA, RNA, and histone methylation. Brain Res. 2025;1865:149852. 40691927 10.1016/j.brainres.2025.149852

[13] Zhang J, et al. Neuromodulatory role and therapeutic potential of N6-methyladenosine RNA methylation in neurodegenerative diseases. Neural Regen Res. 2025. 10.4103/NRR.NRR-D-24-01648 PMC13211820 40618260

[14] Dominissini D, et al. Topology of the human and mouse m6A RNA methylomes revealed by m6A-seq. Nature. 2012;485(7397):201–6. 22575960 10.1038/nature11112

[15] Gilbert WV, Nachtergaele S. mRNA regulation by RNA modifications. Annu Rev Biochem. 2023;92:175–98. 37018844 10.1146/annurev-biochem-052521-035949 PMC11192554

[16] Alzuri SE, et al. Role of miR-124-3p in regulatory mechanisms of Gpm6a expression in the hippocampus of chronically stressed rats. J Neurochem. 2023;165(4):603–21. 36943192 10.1111/jnc.15810

[17] Huang J, et al. m(6)A-modified lincRNA Dubr is required for neuronal development by stabilizing YTHDF1/3 and facilitating mRNA translation. Cell Rep. 2022;41(8):111693. 36417851 10.1016/j.celrep.2022.111693

[18] Zhao BS, Roundtree IA, He C. Post-transcriptional gene regulation by mRNA modifications. Nat Rev Mol Cell Biol. 2017;18(1):31–42. 27808276 10.1038/nrm.2016.132 PMC5167638

[19] Zaccara S, Ries RJ, Jaffrey SR. Reading, writing and erasing mRNA methylation. Nat Rev Mol Cell Biol. 2019;20(10):608–24. 31520073 10.1038/s41580-019-0168-5

[20] Han M, et al. Abnormality of m6A mRNA methylation is involved in Alzheimer’s disease. Front Neurosci. 2020;14:98. 32184705 10.3389/fnins.2020.00098 PMC7058666

[21] Deng J, et al. N6-methyladenosine demethylase FTO regulates synaptic and cognitive impairment by destabilizing PTEN mRNA in hypoxic-ischemic neonatal rats. Cell Death Dis. 2023;14(12):820. 38092760 10.1038/s41419-023-06343-5 PMC10719319

[22] Yu F, et al. Post-translational modification of RNA m6A demethylase ALKBH5 regulates ROS-induced DNA damage response. Nucleic Acids Res. 2021;49(10):5779–97. 34048572 10.1093/nar/gkab415 PMC8191756

[23] Li B, et al. FTO-dependent m(6)A modification of Plpp3 in circSCMH1-regulated vascular repair and functional recovery following stroke. Nat Commun. 2023;14(1):489. 36717587 10.1038/s41467-023-36008-y PMC9886939

[24] Yankova E, et al. Small-molecule inhibition of METTL3 as a strategy against myeloid leukaemia. Nature. 2021;593(7860):597–601. 33902106 10.1038/s41586-021-03536-w PMC7613134

[25] Jiang L, et al. Interaction of tau with HNRNPA2B1 and N(6)-methyladenosine RNA mediates the progression of tauopathy. Mol Cell. 2021;81(20):4209–e422712. 34453888 10.1016/j.molcel.2021.07.038 PMC8541906

[26] Sevgi M, et al. An obesity-predisposing variant of the FTO gene regulates D2R-dependent reward learning. J Neurosci. 2015;35(36):12584–92. 26354923 10.1523/JNEUROSCI.1589-15.2015 PMC6605390

[27] He H, et al. METTL14 is decreased and regulates m(6) a modification of alpha-synuclein in Parkinson’s disease. J Neurochem. 2023;166(3):609–22. 37309980 10.1111/jnc.15882

[28] Perlegos AE, et al. Mettl3-dependent m(6)A modification attenuates the brain stress response in Drosophila. Nat Commun. 2022;13(1):5387. 36104353 10.1038/s41467-022-33085-3 PMC9474545

[29] Zhong X, et al. METTL14/YTHDC1-mediated m6A modification in hippocampus improves pentylenetetrazol-induced acute seizures. Mol Neurobiol. 2024;61(12):10979–91. 38814536 10.1007/s12035-024-04252-y

[30] Wang LJ, et al. Wilms’ tumour 1-associating protein inhibits endothelial cell angiogenesis by m6A-dependent epigenetic silencing of desmoplakin in brain arteriovenous malformation. J Cell Mol Med. 2020;24(9):4981–91. 32281240 10.1111/jcmm.15101 PMC7205785

[31] Lin X, et al. KIAA1429 promotes tumorigenesis and gefitinib resistance in lung adenocarcinoma by activating the JNK/ MAPK pathway in an m(6)A-dependent manner. Drug Resist Updat. 2023;66:100908. 36493511 10.1016/j.drup.2022.100908

[32] Leismann J, et al. The 18S ribosomal RNA m(6) A methyltransferase Mettl5 is required for normal walking behavior in Drosophila. EMBO Rep. 2020;21(7):e49443. 32350990 10.15252/embr.201949443 PMC7332798

[33] Shen D, et al. Detailed resume of RNA m(6)A demethylases. Acta Pharm Sin B. 2022;12(5):2193–205. 35646549 10.1016/j.apsb.2022.01.003 PMC9136571

[34] Meyer KD, Jaffrey SR. The dynamic epitranscriptome: N6-methyladenosine and gene expression control. Nat Rev Mol Cell Biol. 2014;15(5):313–26. 24713629 10.1038/nrm3785 PMC4393108

[35] Chang R, et al. The hippocampal FTO-BDNF-TrkB pathway is required for novel object recognition memory reconsolidation in mice. Transl Psychiatry. 2023;13(1):349. 37963912 10.1038/s41398-023-02647-4 PMC10645923

[36] Walters BJ, et al. The role of The RNA demethylase FTO (fat mass and obesity-associated) and mRNA methylation in hippocampal memory formation. Neuropsychopharmacology. 2017;42(7):1502–10. 28205605 10.1038/npp.2017.31 PMC5436121

[37] Song J, et al. Increased m(6)A modification of BDNF mRNA via FTO promotes neuronal apoptosis following aluminum-induced oxidative stress. Environ Pollut. 2024;349:123848. 38548149 10.1016/j.envpol.2024.123848

[38] Jia G, et al. N6-methyladenosine in nuclear RNA is a major substrate of the obesity-associated FTO. Nat Chem Biol. 2011;7(12):885–7. 22002720 10.1038/nchembio.687 PMC3218240

[39] Shen W, et al. FTO overexpression inhibits apoptosis of hypoxia/reoxygenation-treated myocardial cells by regulating m6A modification of Mhrt. Mol Cell Biochem. 2021;476(5):2171–9. 33548009 10.1007/s11010-021-04069-6

[40] Chokkalla AK, et al. Cerebroprotective role of N(6)-methyladenosine demethylase FTO (fat mass and obesity-associated protein) after experimental stroke. Stroke. 2023;54(1):245–54. 36321453 10.1161/STROKEAHA.122.040401 PMC10250008

[41] De La Martinez B, et al. Modifying the m(6)A brain methylome by ALKBH5-mediated demethylation: a new contender for synaptic tagging. Mol Psychiatry. 2021;26(12):7141–53. 34663904 10.1038/s41380-021-01282-z PMC8872986

[42] Chang Y, et al. N(6)-methyladenosine RNA modification of glutamatergic neurons is associated with contextual fear discrimination. Physiol Behav. 2022;248:113741. 35167878 10.1016/j.physbeh.2022.113741

[43] Qu M, et al. High glucose induces tau hyperphosphorylation in hippocampal neurons via inhibition of ALKBH5-mediated Dgkh m(6)A demethylation: a potential mechanism for diabetic cognitive dysfunction. Cell Death Dis. 2023;14(6):385. 37385994 10.1038/s41419-023-05909-7 PMC10310746

[44] Guo F, et al. Astrocytic ALKBH5 in stress response contributes to depressive-like behaviors in mice. Nat Commun. 2024;15(1):4347. 38773146 10.1038/s41467-024-48730-2 PMC11109195

[45] Du T, et al. RNA demethylase Alkbh5 is widely expressed in neurons and decreased during brain development. Brain Res Bull. 2020;163:150–9. 32717204 10.1016/j.brainresbull.2020.07.018

[46] Shi H, et al. m(6)A facilitates hippocampus-dependent learning and memory through YTHDF1. Nature. 2018;563(7730):249–53. 30401835 10.1038/s41586-018-0666-1 PMC6226095

[47] Zhuang M, et al. The m6A reader YTHDF1 regulates axon guidance through translational control of Robo3.1 expression. Nucleic Acids Res. 2019;47(9):4765–77. 30843071 10.1093/nar/gkz157 PMC6511866

[48] Yu J, et al. The m(6) a readers YTHDF1 and YTHDF2 Synergistically control cerebellar parallel fiber growth by regulating local translation of the key Wnt5a signaling components in Axons. Adv Sci (Weinh). 2021;8(22):e2101329. 34643063 10.1002/advs.202101329 PMC8596126

[49] Xu P, et al. Deoxynivalenol induces m(6)A-mediated upregulation of p21 and growth arrest of mouse hippocampal neuron cells in vitro. Cell Biol Toxicol. 2024;40(1):41. 38833095 10.1007/s10565-024-09872-7 PMC11150311

[50] Niu F, et al. m(6)A regulation of cortical and retinal neurogenesis is mediated by the redundant m(6)A readers YTHDFs. iScience. 2022;25(9):104908. 36039295 10.1016/j.isci.2022.104908 PMC9418916

[51] Zhu XJ, et al. Single-cell sequencing analysis reveals the essential role of the m (6)A reader YTHDF1 in retinal visual function by regulating TULP1 and DHX38 translation. Zool Res. 2025;46(2):429–45. 40116022 10.24272/j.issn.2095-8137.2024.399 PMC12000125

[52] Frye M, et al. RNA modifications modulate gene expression during development. Science. 2018;361(6409):1346–9. 30262497 10.1126/science.aau1646 PMC6436390

[53] Huang H, et al. Recognition of RNA N(6)-methyladenosine by IGF2BP proteins enhances mRNA stability and translation. Nat Cell Biol. 2018;20(3):285–95. 29476152 10.1038/s41556-018-0045-z PMC5826585

[54] Deng Y, et al. Identification of the function and mechanism of m6A reader IGF2BP2 in Alzheimer’s disease. Aging. 2021;13(21):24086–100. 34705667 10.18632/aging.203652 PMC8610118

[55] Zhang H, et al. SUMOylation modification of FTO facilitates oxidative damage response of arsenic by IGF2BP3 in an m6A-dependent manner. J Hazard Mater. 2024;472:134440. 38723480 10.1016/j.jhazmat.2024.134440

[56] Zhao Y, et al. IGF2BP2-Shox2 axis regulates hippocampal-neuronal senescence to alleviate microgravity-induced recognition disturbance. iScience. 2024;27(6):109917. 38812544 10.1016/j.isci.2024.109917 PMC11134919

[57] Wu R, et al. A novel m(6)A reader Prrc2a controls oligodendroglial specification and myelination. Cell Res. 2019;29(1):23–41. 30514900 10.1038/s41422-018-0113-8 PMC6318280

[58] Brandao-Teles C, et al. The roles of hnRNP family in the brain and brain-related disorders. Mol Neurobiol. 2024;61(6):3578–95. 37999871 10.1007/s12035-023-03747-4

[59] Liu J, et al. A METTL3-METTL14 complex mediates mammalian nuclear RNA N6-adenosine methylation. Nat Chem Biol. 2014;10(2):93–5. 24316715 10.1038/nchembio.1432 PMC3911877

[60] Li Z, et al. FTO plays an oncogenic role in acute myeloid leukemia as a N(6)-methyladenosine RNA demethylase. Cancer Cell. 2017;31(1):127–41. 28017614 10.1016/j.ccell.2016.11.017 PMC5234852

[61] Roy B, Ochi S, Dwivedi Y. M6A RNA methylation-based epitranscriptomic modifications in plasticity-related genes via miR-124-C/EBPalpha-FTO-transcriptional axis in the hippocampus of learned helplessness rats. Int J Neuropsychopharmacol. 2022;25(12):1037–49. 36161325 10.1093/ijnp/pyac068 PMC9743968

[62] Zaccara S, Jaffrey SR. A unified model for the function of YTHDF proteins in regulating m(6)A-modified mRNA. Cell. 2020;181(7):1582–e159518. 32492408 10.1016/j.cell.2020.05.012 PMC7508256

[63] Shi H, et al. YTHDF3 facilitates translation and decay of N(6)-methyladenosine-modified RNA. Cell Res. 2017;27(3):315–28. 28106072 10.1038/cr.2017.15 PMC5339834

[64] Liu J, et al. The RNA m(6)A reader YTHDC1 silences retrotransposons and guards ES cell identity. Nature. 2021;591(7849):322–6. 33658714 10.1038/s41586-021-03313-9

[65] Chang M et al. Region-specific RNA m(6)A methylation represents a new layer of control in the gene regulatory network in the mouse brain. Open Biol. 2017;7(9). 10.1098/rsob.170166 PMC5627058 28931651

[66] Meyer KD, et al. Comprehensive analysis of mRNA methylation reveals enrichment in 3’ UTRs and near stop codons. Cell. 2012;149(7):1635–46. 22608085 10.1016/j.cell.2012.05.003 PMC3383396

[67] Yen YP, Chen JA. The m(6)A epitranscriptome on neural development and degeneration. J Biomed Sci. 2021;28(1):40. 34039354 10.1186/s12929-021-00734-6 PMC8157406

[68] You S, et al. Research progress on the role of RNA m6A modification in glial cells in the regulation of neurological diseases. Biomolecules. 2022;12(8):1158. 36009052 10.3390/biom12081158 PMC9405963

[69] Wu X, et al. The m(6)A methyltransferase METTL3 drives neuroinflammation and neurotoxicity through stabilizing BATF mRNA in microglia. Cell Death Differ. 2025;32(1):100–17. 38902548 10.1038/s41418-024-01329-y PMC11742445

[70] Song L, et al. Biological functions of the m6A reader YTHDF2 and its role in central nervous system disorders. Biochem Pharmacol. 2024;230(Pt 1):116576. 39424201 10.1016/j.bcp.2024.116576

[71] Chen J, et al. m(6)A regulates neurogenesis and neuronal development by modulating histone methyltransferase Ezh2. Genomics Proteom Bioinf. 2019;17(2):154–68. 10.1016/j.gpb.2018.12.007 PMC6620265 31154015

[72] Li L, et al. Fat mass and obesity-associated (FTO) protein regulates adult neurogenesis. Hum Mol Genet. 2017;26(13):2398–411. 28398475 10.1093/hmg/ddx128 PMC6192412

[73] Qu W, et al. m(6)A Modification involves in enriched environment-induced neurogenesis and cognition enhancement. Front Cell Dev Biol. 2022;10:903179. 35721485 10.3389/fcell.2022.903179 PMC9201454

[74] Zhang F, et al. m(6)A/YTHDF2-mediated mRNA decay targets TGF-beta signaling to suppress the quiescence acquisition of early postnatal mouse hippocampal NSCs. Cell Stem Cell. 2025;32(1):144–56. e8. 39476834 10.1016/j.stem.2024.10.002 PMC11698649

[75] Li M, et al. Ythdf2-mediated m(6)A mRNA clearance modulates neural development in mice. Genome Biol. 2018;19(1):69. 29855337 10.1186/s13059-018-1436-y PMC5984442

[76] Flamand MN, Meyer KD. m6A and YTHDF proteins contribute to the localization of select neuronal mRNAs. Nucleic Acids Res. 2022;50(8):4464–83. 35438793 10.1093/nar/gkac251 PMC9071445

[77] Lisman J, Schulman H, Cline H. The molecular basis of CaMKII function in synaptic and behavioural memory. Nat Rev Neurosci. 2002;3(3):175–90. 11994750 10.1038/nrn753

[78] Yu J, et al. Dynamic m6A modification regulates local translation of mRNA in axons. Nucleic Acids Res. 2018;46(3):1412–23. 29186567 10.1093/nar/gkx1182 PMC5815124

[79] Ries RJ, et al. m(6)A enhances the phase separation potential of mRNA. Nature. 2019;571(7765):424–8. 31292544 10.1038/s41586-019-1374-1 PMC6662915

[80] Broix L, et al. m(6)A RNA methylation-mediated control of global APC expression is required for local translation of beta-actin and axon development. Cell Rep. 2025;44(6):p115727. 10.1016/j.celrep.2025.115727 40402742

[81] Weng YL, et al. Epitranscriptomic m(6)A regulation of axon regeneration in the adult mammalian nervous system. Neuron. 2018;97(2):313–e3256. 29346752 10.1016/j.neuron.2017.12.036 PMC5777326

[82] Haigis MC, Yankner BA. The aging stress response. Mol Cell. 2010;40(2):333–44. 20965426 10.1016/j.molcel.2010.10.002 PMC2987618

[83] Perlegos AE, Byrns CN, Bonini NM. Cell type-specific regulation of m(6) a modified RNAs in the aging Drosophila brain. Aging Cell. 2024;23(3):e14076. 38205931 10.1111/acel.14076 PMC10928574

[84] Tegowski M, et al. Single-cell m(6)A profiling in the mouse brain uncovers cell type-specific RNA methylomes and age-dependent differential methylation. Nat Neurosci. 2024;27(12):2512–20. 39317796 10.1038/s41593-024-01768-3 PMC11614689

[85] Huang H, et al. The N6-methyladenosine RNA landscape in the aged mouse hippocampus. Aging Cell. 2023;22(1):e13755. 36495001 10.1111/acel.13755 PMC9835576

[86] Castro-Hernandez R, et al. Conserved reduction of m(6)A RNA modifications during aging and neurodegeneration is linked to changes in synaptic transcripts. Proc Natl Acad Sci U S A. 2023;120(9):e2204933120. 36812208 10.1073/pnas.2204933120 PMC9992849

[87] Bennett EL, et al. Chemical and anatomical plasticity brain. Science. 1964;146(3644):610–9. 14191699 10.1126/science.146.3644.610

[88] Hernandez PJ, Abel T. The role of protein synthesis in memory consolidation: progress amid decades of debate. Neurobiol Learn Mem. 2008;89(3):293–311. 18053752 10.1016/j.nlm.2007.09.010 PMC2745628

[89] Widagdo J, et al. Experience-dependent accumulation of N6-methyladenosine in the prefrontal cortex is associated with memory processes in mice. J Neurosci. 2016;36(25):6771–7. 27335407 10.1523/JNEUROSCI.4053-15.2016 PMC4916251

[90] Gilbert CD, Li W. Adult visual cortical plasticity. Neuron. 2012;75(2):250–64. 22841310 10.1016/j.neuron.2012.06.030 PMC3408614

[91] Koyano KW, et al. Progressive neuronal plasticity in primate visual cortex during stimulus familiarization. Sci Adv. 2023;9(12):eade4648. 36961903 10.1126/sciadv.ade4648 PMC10038346

[92] Senna I, et al. Development of multisensory integration following prolonged early-onset visual deprivation. Curr Biol. 2021;31(21):4879–e48856. 34534443 10.1016/j.cub.2021.08.060

[93] Dragoi V, Rivadulla C, Sur M. Foci of orientation plasticity in visual cortex. Nature. 2001;411(6833):80–6. 11333981 10.1038/35075070

[94] Wang HF, et al. Hearing impairment is associated with cognitive decline, brain atrophy and tau pathology. EBioMedicine. 2022;86:104336. 36356475 10.1016/j.ebiom.2022.104336 PMC9649369

[95] Kurioka T, Mogi S, Yamashita T. Decreasing auditory input induces neurogenesis impairment in the hippocampus. Sci Rep. 2021;11(1):423. 33432038 10.1038/s41598-020-80218-z PMC7801596

[96] Zhou X, et al. Comprehensive analysis of N6-methyladenosine-related RNA methylation in the mouse hippocampus after acquired hearing loss. BMC Genomics. 2023;24(1):577. 37759187 10.1186/s12864-023-09697-4 PMC10537436

[97] Vuust P, et al. Music in the brain. Nat Rev Neurosci. 2022;23(5):287–305. 35352057 10.1038/s41583-022-00578-5

[98] Van Gool WA, et al. Effect of housing in an enriched environment on the size of the cerebral cortex in young and old rats. Exp Neurol. 1987;96(1):225–32. 3556515 10.1016/0014-4886(87)90185-3

[99] van Gool WA, Mirmiran M. Effects of aging and housing in an enriched environment on sleep-wake patterns in rats. Sleep. 1986;9(2):335–47. 3505733 10.1093/sleep/9.2.335

[100] Quinlan EB, et al. Peer victimization and its impact on adolescent brain development and psychopathology. Mol Psychiatry. 2020;25(11):3066–76. 30542059 10.1038/s41380-018-0297-9

[101] Lupien SJ, et al. Cortisol levels during human aging predict hippocampal atrophy and memory deficits. Nat Neurosci. 1998;1(1):69–73. 10195112 10.1038/271

[102] Cheng T, et al. Physical exercise rescues cocaine-evoked synaptic deficits in motor cortex. Mol Psychiatry. 2021;26(11):6187–97. 34686765 10.1038/s41380-021-01336-2

[103] Chen K, et al. Exercise training improves motor skill learning via selective activation of mTOR. Sci Adv. 2019;5(7):eaaw1888. 31281888 10.1126/sciadv.aaw1888 PMC6609215

[104] Yan L, et al. Physical Exercise Prevented Stress-Induced Anxiety via Improving Brain RNA Methylation. Adv Sci (Weinh). 2022;9(24):e2105731. 35642952 10.1002/advs.202105731 PMC9404392

[105] Liu SJ, et al. Long-term exercise training down-regulates m(6)A RNA demethylase FTO expression in the hippocampus and hypothalamus: an effective intervention for epigenetic modification. BMC Neurosci. 2022;23(1):54. 36163017 10.1186/s12868-022-00742-8 PMC9513931

[106] Shafik AM, et al. N6-methyladenosine dynamics in neurodevelopment and aging, and its potential role in Alzheimer’s disease. Genome Biol. 2021;22(1):17. 33402207 10.1186/s13059-020-02249-z PMC7786910

[107] Zhao F, et al. METTL3-dependent RNA m(6)A dysregulation contributes to neurodegeneration in Alzheimer’s disease through aberrant cell cycle events. Mol Neurodegener. 2021;16(1):70. 34593014 10.1186/s13024-021-00484-x PMC8482683

[108] Chen H, et al. METTL3 confers protection against mitochondrial dysfunction and cognitive impairment in an Alzheimer disease mouse model by upregulating Mfn2 via N6-methyladenosine modification. J Neuropathol Exp Neurol. 2024;83(7):606–14. 38408379 10.1093/jnen/nlae010

[109] Liu Z, et al. The landscape of m6A regulators in multiple brain regions of Alzheimer’s disease. Mol Neurobiol. 2023;60(9):5184–98. 37273154 10.1007/s12035-023-03409-5

[110] Darweesh SKL, et al. Parkinson Matters J Parkinsons Dis. 2018;8(4):495–8. 30149463 10.3233/JPD-181374 PMC6218141

[111] Chen X, et al. Down-regulation of m6A mRNA methylation is involved in dopaminergic neuronal death. ACS Chem Neurosci. 2019;10(5):2355–63. 30835997 10.1021/acschemneuro.8b00657

[112] The Huntington’s Disease Collaborative Research Group. A novel gene containing a trinucleotide repeat that is expanded and unstable on Huntington’s disease chromosomes. Cell. 1993;72(6):971–83. 8458085 10.1016/0092-8674(93)90585-e

[113] Nguyen TB, et al. Aberrant splicing in Huntington’s disease accompanies disrupted TDP-43 activity and altered m6A RNA modification. Nat Neurosci. 2025;28(2):280–92. 39762660 10.1038/s41593-024-01850-w PMC11802453

[114] McMillan M, et al. RNA methylation influences TDP43 binding and disease pathogenesis in models of amyotrophic lateral sclerosis and frontotemporal dementia. Mol Cell. 2023;83(2):219–e2367. 36634675 10.1016/j.molcel.2022.12.019 PMC9899051

[115] Pupak A, et al. Altered m6A RNA methylation contributes to hippocampal memory deficits in Huntington’s disease mice. Cell Mol Life Sci. 2022;79(8):416. 35819730 10.1007/s00018-022-04444-6 PMC9276730

[116] Benesch C, et al. Perioperative neurological evaluation and management to lower the risk of acute stroke in patients undergoing noncardiac, nonneurological surgery: a scientific statement from the American heart association/American stroke association. Circulation. 2021;143(19):e923–46. 33827230 10.1161/CIR.0000000000000968

[117] Zhang F, et al. Regulation of N6-methyladenosine (m6A) RNA methylation in microglia-mediated inflammation and ischemic stroke. Front Cell Neurosci. 2022;16:955222. 35990887 10.3389/fncel.2022.955222 PMC9386152

[118] Su X, et al. The RNA m6A modification might participate in microglial activation during hypoxic-ischemic brain damage in neonatal mice. Hum Genomics. 2023;17(1):78. 37626401 10.1186/s40246-023-00527-y PMC10463984

[119] Liu C, et al. ALKBH5 protects against stroke by reducing endoplasmic reticulum stress-dependent inflammation injury via the STAT5/PERK/EIF2alpha/CHOP signaling pathway in an m(6)A-YTHDF1-dependent manner. Exp Neurol. 2024;372:114629. 38056583 10.1016/j.expneurol.2023.114629

[120] Li Y, et al. Globally reduced N(6)-methyladenosine (m(6)A) in C9ORF72-ALS/FTD dysregulates RNA metabolism and contributes to neurodegeneration. Nat Neurosci. 2023;26(8):1328–38. 37365312 10.1038/s41593-023-01374-9 PMC11361766

[121] Yang L, et al. Aluminum causes irreversible damage to the development of hippocampal neurons by regulating m6A RNA methylation. Toxicol Lett. 2024;399:34–42. 39009234 10.1016/j.toxlet.2024.07.908

[122] Bai L, et al. m6A demethylase FTO regulates dopaminergic neurotransmission deficits caused by Arsenite. Toxicol Sci. 2018;165(2):431–46. 29982692 10.1093/toxsci/kfy172

[123] Weekley BH, Ahmed NI, Maze I. Elucidating neuroepigenetic mechanisms to inform targeted therapeutics for brain disorders. iScience. 2025;28(3):112092. 40160416 10.1016/j.isci.2025.112092 PMC11951040

[124] Hu B, et al. Rewired m6A of promoter antisense RNAs in Alzheimer’s disease regulates neuronal genes in 3D nucleome. Nat Commun. 2025;16(1):5251. 40480976 10.1038/s41467-025-60378-0 PMC12144123

[125] Liu X, et al. Tailoring and reversing m6A editing with sequential RNA bioorthogonal chemistry. Nucleic Acids Res. 2025;53(7):gkaf283. 40219967 10.1093/nar/gkaf283 PMC11992675

[126] Feng S, Koziol MJ. Unraveling brain complexity: from single-cell to spatial m(6)A technologies. Trends Genet. 2025. 10.1016/j.tig.2025.06.010 40803945

